# Sensor-Efficient Estimation of Roll Misalignment via Side-to-Side Tension Differences in Roll-to-Roll Polymer Film Processing

**DOI:** 10.3390/polym17212907

**Published:** 2025-10-30

**Authors:** Junyoung Yun, Sangbin Lee, Jeongwon Jang, Mingi Kim, Chanwoo Kim, Changwoo Lee

**Affiliations:** 1 Department of Mechanical Design and Production Engineering, Konkuk University, 120 Neungdong-ro, Gwangjin-gu, Seoul 05023, Republic of Korea; jun980520@konkuk.ac.kr (J.Y.); tkdqls2052@konkuk.ac.kr (S.L.); jpjang0305@konkuk.ac.kr (J.J.); gxkr508@konkuk.ac.kr (M.K.); kcw9817@konkuk.ac.kr (C.K.); 2 Department of Mechanical Engineering, Konkuk University, 120 Neungdong-ro, Gwangjin-gu, Seoul 05029, Republic of Korea

**Keywords:** misalignment detection, polymer film alignment, R2R processing, side-to-side tension difference, web handling dynamics

## Abstract

Roll misalignment in roll-to-roll (R2R) processes is a critical cause of lateral displacement and tension imbalance, leading to dimensional instability and surface defects in polymer films. Conventional analyses based on beam or camber models often require complex calibration and additional sensing, which can limit their applicability in real-time production environments. This study introduces a diagnostic approach that estimates web misalignment directly from side-to-side tension differences measured in roll-to-roll (R2R) systems. The method eliminates the need for additional sensors and complex geometric calibration, simplifying system setup. The correlation between tension imbalance, lateral displacement, and the equivalent misalignment angle was experimentally established. Our approach produced accurate predictions across various process conditions, including different roll misalignments and applied tensions, and we found that reducing tension “hunting” further enhances prediction stability. This study demonstrates that the proposed tension-based approach can complement existing systems and reduce the reliance on complex external sensing for diagnostic checks of misalignment. By simplifying alignment diagnostics, the method provides a practical route to enhance process setup, reduce downtime, and improve the uniformity of polymer films in continuous manufacturing.

## 1. Introduction

R2R processing has become a cornerstone technology for the large-area, continuous manufacturing of polymer films, enabling applications ranging from functional coatings and barrier packaging to flexible electronics, energy harvesting devices, and biomedical substrates [1,2,3,4,5,6,7,8,9]. Compared to batch processing, R2R offers scalability, throughput, and cost-effectiveness, which are critical for both commodity and high-value polymer products [10,11,12,13,14,15,16]. However, maintaining film quality during continuous production remains a major challenge. Web instabilities such as lateral displacement, edge waviness, and tension imbalance can compromise the dimensional stability and surface integrity of polymer films [17,18,19,20,21]. Among these, roll misalignment is recognized as one of the most persistent and detrimental factors, as it induces side-to-side tension variation across the web and leads to the accumulation of significant lateral error during transport [22,23,24,25,26].

Misalignment not only introduces geometric deviations but also degrades functional performance when films are used in multilayer stacks, laminated composites, or patterned devices [27,28,29]. Even small angular errors in roll setup can propagate into coating non-uniformity and layer-to-layer registration drift in printed/laminated architectures, where tight overlay is required for high-value applications such as displays and photovoltaics [30,31,32,33]. Recent studies in R2R printed electronics quantify that registration accuracy is highly sensitive to web tension history and thermo-mechanical distortion, underscoring the vulnerability of multilayer processes to misalignment-induced lateral displacement [34,35,36].

In addition to its mechanical implications, roll misalignment can significantly influence the physical characteristics of polymer films. Uneven lateral tension generates localized variations in strain and residual stress, which can lead to thickness non-uniformity, surface waviness, or edge deformation. These effects directly impact the dimensional stability and surface quality of the film, particularly in multilayer coatings and laminated composites, where even small strain mismatches can cause interfacial defects or wrinkling. Therefore, accurate assessment of misalignment is not only a matter of mechanical alignment but also essential for preserving the intrinsic properties and functional reliability of polymer films during continuous processing.

To address these issues, numerous analytical and numerical models for lateral web dynamics have been developed [37,38,39,40]. Classical beam-based formulations capture lateral motion under tension and boundary conditions, while extensions consider geometric imperfections (e.g., camber) and multi-body guiding dynamics relevant to industrial lines [22,23]. These models have advanced physical understanding and control design, yet their deployment on production equipment is often constrained by the need for detailed parameter identification, precise geometric calibration, and auxiliary displacement/optical sensing. This limits their real-time applicability.

Industrial practice often relies on guide rolls, referred to as edge position control (EPC) systems, as a corrective solution to misalignment. EPCs monitor web edge position and adjust roll orientation or guide mechanisms to maintain alignment [41,42]. While widely adopted, EPCs present several drawbacks. They increase system cost and complexity, require space for actuators and sensors, and, in some high-speed or thin-film processes, may lack sufficient responsiveness to prevent accumulated error. Moreover, EPCs are corrective rather than diagnostic, meaning they act after misalignment occurs rather than identifying its root cause. For polymer films with high sensitivity to mechanical stress, EPC overcorrection can also cause strain deviation across the web, introducing new defects such as edge damage or local wrinkling [43,44,45].

In this context, there is growing interest in diagnostic approaches that leverage existing sensor infrastructure to assess misalignment directly. This study proposes a simple yet reliable approach that uses side-to-side tension difference as a proxy for roll misalignment in polymer film handling, eliminating the need for complex, external sensing. Unlike conventional methods, our approach does not require additional instrumentation beyond standard load cell sensors that are already present in industrial R2R lines. This feature is particularly advantageous during system setup, roll replacement, or product transitions, where rapid alignment verification is critical. By converting tension imbalance into an indicator of misalignment angle, the proposed approach provides a sensor-efficient pathway to support process setup, reduce reliance on EPC, and enhance alignment diagnostics in continuous polymer film manufacturing.

## 2. Theoretical Background and Conceptual Framework

### 2.1. Fundamental Theory of Lateral Web Dynamics

**Figure 1:**
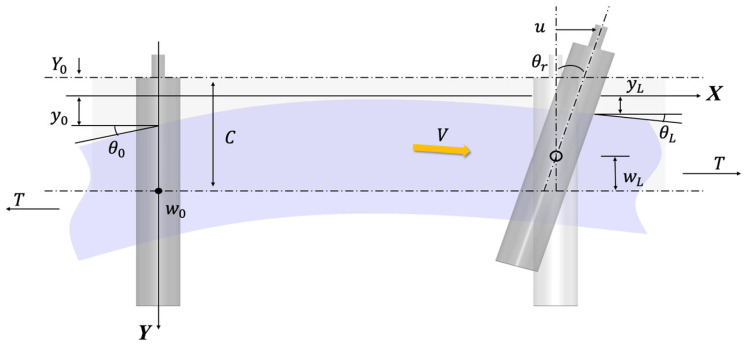
Boundary condition of Shelton’s model.

The analysis of a web’s lateral motion is a foundational aspect of understanding its behavior in a process line. In a typical R2R process line, a polymer web is continuously transported under longitudinal tension between rollers. The web behaves mechanically as a slender beam, where lateral motion arises from the interplay between applied tension, bending stiffness, and roller boundary conditions. As shown in Figure 1, classical beam theory provides the foundation for deriving the governing equation of the web’s lateral displacement.

The linear ordinary differential equation for the motion of the web traveling in the direction of V is given by [13]:

(1) $$\frac{\partial^{4}y}{\partial x^{4}}-K^{2}\frac{\partial^{2}y}{\partial x^{2}}=0,where\;K^{2}=\frac{T}{EI\left(1+nT/AG\right)}.$$

Here, EI represents the bending stiffness, which resists bending deformation, while T is the tension applied to the web. The term K is a constant that accounts for additional factors such as shear and bending effects, where n, A, and G denote the correction factor for actual deflection, cross-sectional area of web, and shear modulus, respectively. This equation establishes the fundamental relationship between the internal forces and the resulting lateral deformation.

The general solution of Equation (1) can be written as:

(2) $$y=C_{1}\sinh\left(Kx\right)+C_{2}\cosh\left(Kx\right)+C_{3}x+C_{4},$$

where $C_{i}$ are constants determined by the roller boundary conditions. Assuming no slip and planar roller action, the boundary conditions are:

(3) $$y\left(0\right)=y_{0},\theta \left(0\right)=\theta_{0},y\left(L\right)=y_{L},\theta \left(L\right)=\theta_{L}.$$

Applying these conditions yields the curvature at the downstream roller:

(4) $$\frac{\partial^{2}y}{\partial x^{2}}{|}_{x=L}=\frac{f_{1}\left(KL\right)}{L_{2}}\left(y_{0}-y_{L}\right)+\frac{f_{2}(KL)}{L}\theta_{L}+\frac{f_{3}(KL)}{L}\theta_{0},$$

where:

$$\begin{array}{c} f_{1}\left(KL\right)={\left(KL\right)}^{2}\left\{coh\left(KL\right)-1\right\}/\{KLsinh\left(KL\right)-2\cosh\left(KL\right)+2\}\\ f_{2}\left(KL\right)=KL^{2}\left\{KLcosh\left(KL\right)-\sinh\left(KL\right)\right\}/\{KLsinh\left(KL\right)-2\cosh\left(KL\right)+2\}\\ f_{3}\left(KL\right)=KL\left\{\sinh\left(KL\right)-KL\right\}/KLsinh\left(KL\right)-2\cosh\left(KL\right)+2\} \end{array}$$

These expressions establish the dependence of web curvature on roller misalignment and span geometry, thereby linking the web’s lateral dynamics to roller motion. Substitution of the static beam solution into the dynamic formulation yields a second-order differential equation, which serves as the basis for subsequent analysis and control system design.

### 2.2. Concept of Side-to-Side Tension Imbalance as a Proxy for Misalignment

Roll misalignment in R2R processes is a critical cause of lateral displacement and tension imbalance, which can lead to dimensional instability and surface defects in polymer films. This study utilizes a beam-based camber model to analyze these effects. By integrating the concept of side-to-side tension imbalance, the following derivations and physical explanations demonstrate how this readily available measurement in industrial R2R systems can be used for real-time monitoring and simplified alignment diagnostics.

#### 2.2.1. Modeling of Lateral Dynamics of Cambered Web

**Figure 2:**
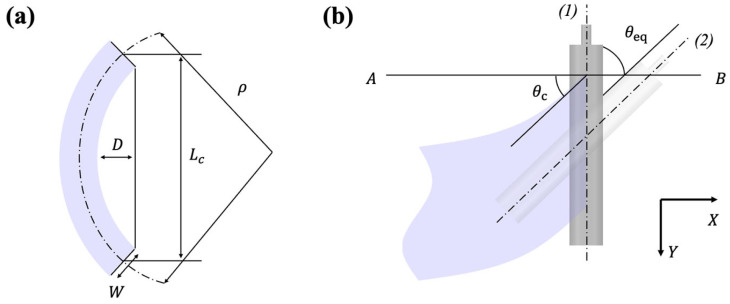
Schematics for (**a**) measurement of camber and (**b**) camber-induced equivalent angle.

Modeling the lateral dynamics of a cambered web requires a static analysis to define its geometric characteristics, followed by a description of its response under applied tension. Camber is defined as the radius of curvature of the web in its un-tensioned state on a flat surface, as illustrated in Figure 2a. The radius ρ, can be obtained from the span length $L_{c}$ and the maximum edge deviation D, as expressed in:

(5) $$\rho =\frac{L_{c}^{2}}{8D}.$$

The lateral response of a cambered web is governed by boundary conditions distinct from those of a straight web. In the initial state, the cambered web has constant curvature −1/ρ, with zero displacement and slope at the upstream end, as given by:

(6) $$y\left(0\right)=0,\theta \left(0\right)=0,y\left(L\right)=0,y\prime \prime (L)=-1/\rho .$$

Once the web laterally shifts, referred to as “walked”, and reaches a new equilibrium, it aligns perpendicularly to the downstream roller, leading to a zero-slope condition (∂y/∂x=0). Applying these boundary conditions to the general solution of Equation (2) yields the initial and steady-state lateral displacement, $y_{ini}$ and $y_{new}$:

(7) $$\begin{array}{cc} y_{ini}\left(x\right)=\frac{1}{\rho }\cdot & \frac{1}{K^{2}(-\sinh\left(KL\right)+cosh(KL)\cdot KL}\\ & \;\;\cdot [-\left(\cosh\left(KL\right)-1\right)\sinh\left(Kx\right)+\left(\sinh\left(KL\right)-KL\right)\cosh\left(Kx\right)+\left(\cosh\left(KL\right)-1\right)x\\ & \;\;-\left(\sinh\left(KL\right)-KL\right)], \end{array}$$

(8) $$y_{new}\left(x\right)=\frac{1}{\rho }\cdot \frac{1}{K^{2}}\left[-\frac{\sinh\left(KL\right)}{\cosh\left(KL\right)-1}\sinh\left(Kx\right)+\cosh\left(Kx\right)+\frac{\sinh\left(KL\right)}{\cosh\left(KL\right)-1}x-1\right].$$

Because camber and steering effects cannot be directly superimposed, an equivalent web angle, illustrated in Figure 2b, is introduced to represent the slope of the cambered web at the downstream roller in its initial state.

(9) $$\theta_{eq}=-\partial y_{ini}/\partial x{|}_{x=L}$$

For comparison, the boundary conditions of a uniform web subjected to a steering angle $\theta_{L}$ at the downstream roller are:

(10) $$y\left(0\right)=0,y^{\prime }\left(0\right)=0,y\left(L\right)=\theta_{L},y^{\prime \prime }\left(L\right)=0$$

Substituting the above into the general solution, Equation (2), gives the lateral displacement at the downstream roller $y_{L}$, in terms of the steering angle $\theta_{L}$:

(11) $$y_{L}=\theta_{L}\frac{\cosh\left(KL\right)KL-sinh(KL)}{K(\cosh\left(KL\right)-1)}.$$

By substituting the equivalent angle ($\theta_{eq}$) from Equation (9) for the steering angle ($\theta_{L}$) in Equation (11), we arrive at Equation (12).

(12) $$y_{L}=\frac{2-2\cosh\left(KL\right)+\sinh\left(KL\right)KL}{\rho K^{2}(\cosh\left(KL\right)-1)}.$$

In summary, the equivalent-angle approach provides a rigorous framework to relate the dynamics of a cambered web to those of a uniform web. By incorporating the equivalent angle as a boundary condition in the governing equation, the established dynamic model for uniform webs can be systematically extended to capture the influence of camber. This formulation serves as a foundation for subsequent analysis in this study.

#### 2.2.2. Physical Explanation and Tension-Based Estimation of Camber

**Figure 3:**
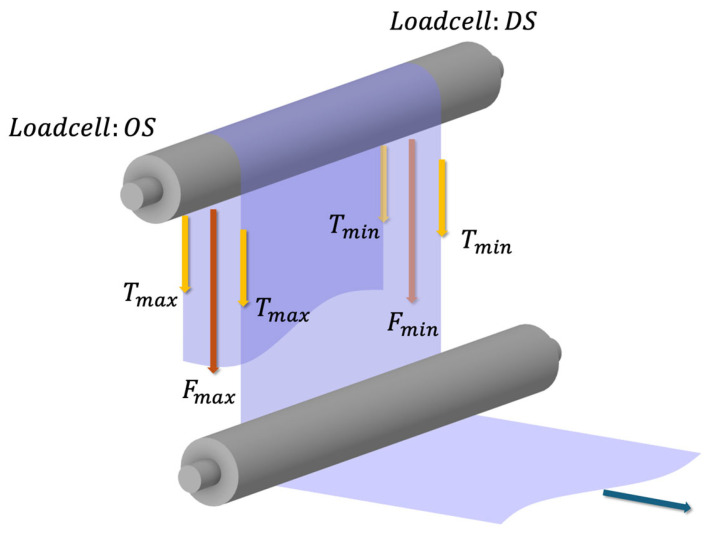
Camber measurement via side-to-side tension imbalance.

The physical link between lateral motion and tension is based on the principle that a web under tension behaves similarly to a beam. With a loadcell setup as illustrated in Figure 3, a misaligned or cambered web experiences a higher tension on its longer edge and a lower tension on its shorter edge. This tension differential creates a net moment.

This relationship is formalized by connecting the moment M, required to straighten a cambered web to its curvature ρ, and subsequently to the web tension T. The final equations are:

(13) $$\frac{1}{\rho }=\frac{M}{EI}=\frac{T_{max}-T_{min}}{6WT}.$$

To clarify the novelty of the theoretical framework, we explicitly distinguish between prior and original contributions. Equations (1)–(4) summarize classical web lateral dynamics derived from Shelton [22,23] and related works [24,25,26]. Equations (5)–(9) introduce the equivalent-angle formulation, which uniquely integrates camber and steering effects for roll misalignment estimation (novel contribution). Finally, Equations (10)–(13) establish a direct relationship between side-to-side tension imbalance and lateral displacement, forming the predictive framework proposed in this study (novel contribution).

For validation of these formulations, representative experimental results are presented in Section 4.1. Relative errors (%) between predicted and measured lateral displacement and equivalent misalignment angle are reported, confirming the numerical accuracy and practical applicability of the proposed method.

These equations demonstrate that the required moment to correct the curvature is proportional to the bending stiffness EI. More importantly, the second equation explicitly shows that the difference between the maximum and minimum tensions, $T_{max}-T_{min}$, is directly proportional to the web’s curvature and its operating tension T, multiplied by the width of web W. This confirms that a measurable side-to-side tension imbalance is a direct and reliable indicator of roll misalignment. By simplifying alignment diagnostics, this sensor-efficient approach enables real-time monitoring without any additional displacement sensing. The derivation from Equations (1)–(13) thus provides a complete theoretical framework for prediction and analysis for the lateral dynamics of a cambered web under varying tension and roller conditions.

### 2.3. Distinction from Prior Methods

Traditional EPC systems for lateral web guidance often rely on dedicated sensors, such as optical or ultrasonic detectors, to measure lateral displacement. While these systems are effective, they introduce additional complexity during setup, requiring sensor installation, wiring, and calibration before production can begin.

The proposed method fundamentally differs by using the side-to-side tension imbalance as a direct proxy for roll misalignment—a signal already available in most industrial R2R systems. By leveraging existing tension sensors, the approach eliminates the need for external hardware and complex calibration, enabling rapid alignment diagnostics during roll setup or product transitions. This sensor-efficient strategy offers a practical and cost-effective route to enhance process efficiency and improve film uniformity in continuous manufacturing.

The preceding sections establish the theoretical formulation connecting side-to-side tension imbalance with lateral displacement and equivalent misalignment angle.

To ensure an objective assessment of the model validity, the following sections (Section 3 and Section 4) describe the independent experimental setup and validation results. This separation clearly distinguishes the analytical development (Equations (1)–(13)) from the empirical verification and enables quantitative evaluation of model accuracy under realistic operating conditions. In contrast to optical or displacement-based EPC systems that demand geometric calibration and dedicated sensors, the proposed ΔT-based method directly infers misalignment from native tension signals, thereby simplifying diagnostics and reducing the need for additional hardware or setup procedures.

## 3. Materials and Methods

### 3.1. Experimental R2R System and Materials

**Figure 4:**
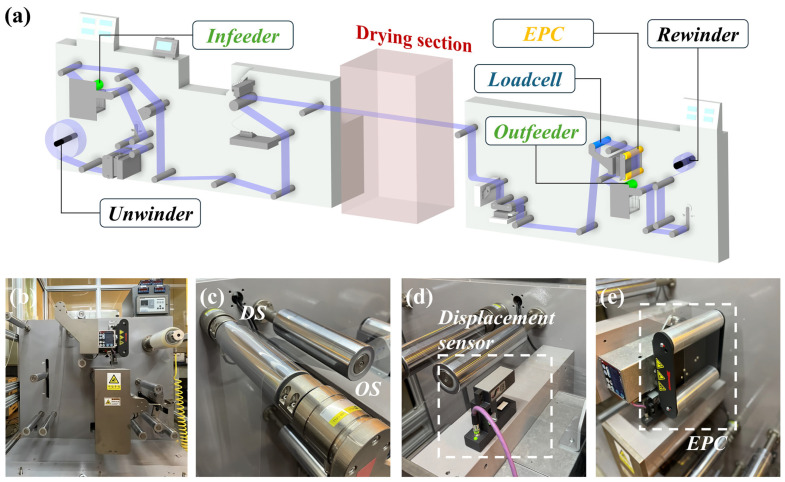
(**a**) Schematics of R2R system, (**b**) outfeeder—unwinder section (**c**) load cell, (**d**) ultrasonic lateral displacement sensor, and (**e**) edge position control unit.

Experiments were conducted on an industrial-scale R2R platform comprising an unwinder, a driven outfeeder, and a rewinder (Figure 4a). The web span between the infeeder and outfeeder roll was designated as the primary region for evaluating lateral dynamics, with particular focus on a 1.5 m section encompassing the roll immediately upstream of the load cell (RTB15, DACELL, Inc., Korea), the load cell itself, and the EPC unit (Figure 4b). Web tension was continuously monitored via load cells (Figure 4c), while lateral displacement was independently validated using an ultrasonic sensor positioned downstream of the span (Figure 4d). The EPC unit enabled controlled roll misalignments for both case studies and model verification (Figure 4e).

To characterize misalignment effects, tension was measured simultaneously at the OS and DS by load-cell sensors mounted within the converting span. The differential tension signal between OS and DS served as the primary diagnostic parameter. All signals were acquired through a data acquisition unit (NI 9239, National Instruments Corp., Austin, TX, USA) at a sampling frequency of 2000 Hz.

Mechanical testing was performed using the same PET film under ambient conditions (22 °C, 30 mm gauge length). The resulting Young’s modulus (3.19 ± 0.08 GPa) and yield stress (110 ± 5 MPa) are consistent with literature values for amorphous PET films [46,47]. In addition to these material properties, Table 1 also lists the key operating parameters applied in the validation experiments, including the line speed and web tension. Sensitivity analysis applying a ±5% variation in E showed less than 2% deviation in predicted displacement and equivalent misalignment angle, confirming that the theoretical model (Equations (1)–(13)) remains robust to small variations in material stiffness.

### 3.2. Experimental Protocols: Controlled Misalignment and Multi-Condition Validation

To examine the sensitivity of lateral tension imbalance to roll misalignment, the EPC was intentionally tilted by prescribed angular offsets in the range of 0.01–0.03°. Each misalignment condition was maintained for a sufficient web length to reach steady-state transport, ensuring that transient effects were excluded from analysis. For statistical reliability, three repeated trials were performed under identical conditions.

To ensure robustness of the proposed estimation approach, multi-condition experiments were performed by varying both the imposed misalignment and the applied web tension level. A factorial design was adopted, combining three roll misalignment angles (0.01°, 0.02°, 0.03°) with three nominal web tension levels (low, medium, high). This resulted in nine distinct case configurations, as summarized in Table 2.

The differential tension signals (ΔT) obtained under these nine operating points were directly compared against lateral displacement measurements from the ultrasonic sensor. By covering a wide range of loading and misalignment conditions, this validation ensured that the estimation method was not restricted to a single nominal configuration but remained effective across diverse process states.

### 3.3. Data Processing and Error Quantification

Acquired tension signals were detrended to remove baseline drift and digitally filtered to suppress high-frequency. The side-to-side tension difference was then calculated as

(14) $$∆T=\left|T_{OS}-T_{DS}\right|$$

where $T_{OS}\;$ and $T_{DS}$ are the OS and DS tensions, respectively. The corresponding lateral displacement from the ultrasonic sensor was synchronized to the same time base for validation.

To evaluate the accuracy of the proposed misalignment estimation, representative-value comparisons were performed. For displacement, the mean predicted displacement ${\hat{y}}_{mean}$, obtained from ∆T using Equations (12) and (13), was compared with the mean measured displacement $y_{mean}$ from the ultrasonic sensor. The relative error (RE) was calculated as

(15) $$RE_{y}=\frac{\left|{\hat{y}}_{mean}-y_{mean}\right|}{y_{mean}}\times 100\%.$$

For equivalent misalignment angle, the mean predicted ${\hat{\theta }}_{eq}$, obtained by mapping displacement through Equation (11), was compared directly with the imposed angle $\theta_{set}$ from the EPC actuator. The relative error for equivalent angle was defined as

(16) $$RE_{\theta }=\frac{\left|{\hat{\theta }}_{eq}-\theta_{set}\right|}{\theta_{set}}\times 100\%.$$

Together, these metrics provide a consistent basis for evaluating the accuracy of both displacement and equivalent misalignment angle across all experimental cases.

## 4. Results and Discussions

### 4.1. Stepwise Application of the Diagnostic Approach

To demonstrate the diagnostic framework, a representative operating condition was selected with medium web tension (T=26.5 N) and a moderate roll misalignment ($\theta_{set}=0.02{}^{\circ}$). The imposed angular offset produced an asymmetric tensile distribution, with OS tension increasing to 28.88 N and DS tension decreasing to 23.28 N.

**Figure 5:**
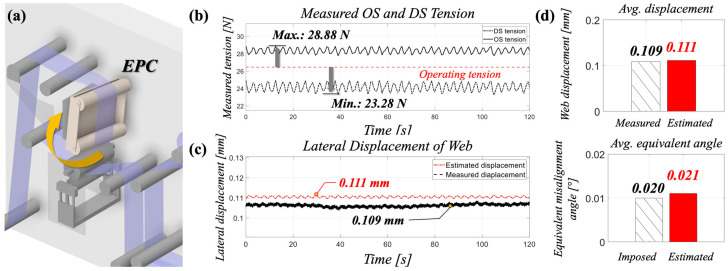
(**a**) Schematics of imposed roll misalignment via EPC, (**b**) Measured OS and DS tension over time, (**c**) the predicted displacement via suggested method and measured displacement with ultrasonic sensor, and (**d**) predicted displacement of web and equivalent misalignment angle via suggested method and measured/imposed values.

Figure 5b illustrates the temporal evolution of the OS and DS tensions. The nominal operating tension is indicated by the red dashed line, around which the two signals diverge due to the imposed misalignment. The OS tension rose above the reference level, reaching a maximum of 28.88 N, while the DS tension decreased to a minimum of 23.28 N. This differential tension ∆T was subsequently processed to estimate the lateral displacement of the web edge using Equations (12) and (13). As shown in Figure 5c, the predicted displacement closely matched the independent measurements obtained from the ultrasonic sensor, with minimal bias across the test interval. The peak displacement values, highlighted by circles in the figure, were 0.111 mm (predicted) and 0.109 mm (measured), corresponding to an absolute error of only 0.002 mm.

Building on this validation, the displacement was further mapped into an equivalent misalignment angle through Equation (11). The estimated $\theta_{eq}\;$ exhibited strong agreement with the imposed angular offset $\theta_{set}$. Figure 5d compares the average displacement and equivalent misalignment angle obtained from the proposed estimation method and the imposed value $\theta_{set}$. The values were closely matched, with displacements of 0.109 mm (measured) and 0.111 mm (estimated), and corresponding equivalent angles of 0.021° (estimated) and 0.020° (imposed).

This agreement demonstrates that the side-to-side tension imbalance, when properly formulated, can serve as a reliable predictor of both lateral displacement and effective misalignment angle. The clear progression from an imposed roll offset to the reconstructed diagnostic variables underscores the feasibility of differential tension as a practical indicator of roll misalignment in polymer film handling. To further reinforce this conclusion, the outcomes are summarized in Table 3 alongside the time-domain comparison in Figure 5. The table presents the measured tension imbalance (∆T), the predicted displacement obtained from Equations (12) and (13), the corresponding ultrasonic sensor measurement, and the equivalent misalignment angle derived from Equation (11). The consistently close agreement across these parameters highlights the robustness of differential tension as a diagnostic metric, and provides a foundation for broader validation under varying operating conditions discussed in the following subsections.

The similarity between predicted and measured values validates the feasibility of estimating misalignment directly from tension data. Building on this reference case, the subsequent sections systematically investigate the effects of varying operating tension and imposed angular offsets to assess the generality of the proposed diagnostic framework.

### 4.2. Multi-Condition General Validation

**Figure 6:**
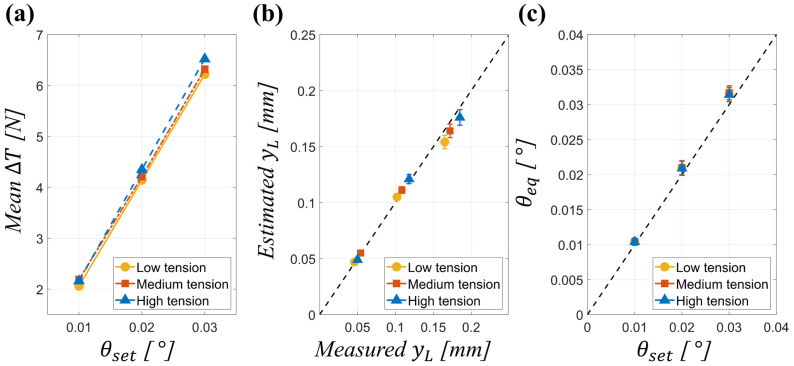
(**a**) Mean differential tension (∆T) as a function of imposed misalignment angle for different web tensions, (**b**) predicted versus measured web-edge displacement, and (**c**) reconstructed equivalent misalignment angle versus imposed EPC angle.

To extend the reference case validation Section 4.1, multi-condition experiments were conducted across nine test cases by combining three imposed roll misalignment angles (0.01°, 0.02°, 0.03°) with three web tension levels (low, medium, and high). The objective was to confirm the generality of the proposed framework in predicting lateral displacement and equivalent misalignment angle under varying operating conditions. To ensure statistical reliability, each experimental configuration summarized in Table 2 was repeated five times (n = 5) under identical process conditions. For each trial we recorded OS and DS tensions and the ultrasonic displacement trace; reported values in Table 4 and Table 5 are the arithmetic mean ± Standard Deviation (SD) across the five repetitions. Relative error values (%RE) are reported as the mean ± SD and were computed as described in Equations (15) and (16). Error bars corresponding to ±1 SD have been added to Figure 6b,c.

Figure 6 provides a comprehensive visualization of the validation results for the proposed diagnostic framework across varying tension levels and imposed misalignment angles. In Figure 6a, the mean differential tension (∆T) is plotted against the imposed misalignment angle ($\theta_{set}$) for low, medium, and high-tension conditions, revealing a clear linear relationship that is consistent across all tension levels. This confirms the expected mechanical behavior whereby ∆T scales proportionally with $\theta_{set}$. Figure 6b presents the reconstructed lateral displacement at the web edge ($y_{L}$) compared with experimental measurements, showing close agreement and highlighting the framework’s predictive capability. Figure 6c depicts the reconstructed equivalent misalignment angles ($\theta_{eq}$) as a function of the imposed EPC angles, with all cases lying near the optimal prediction line, indicating that the model robustly captures misalignment effects under different operating tensions.

The quantitative data summarized in Table 4 support these observations. The mean differential tension (∆T) exhibits an almost linear scaling with respect to the imposed misalignment angle $\theta_{set}$. For instance, under low-tension conditions, ∆T increased from 2.06 N at $\theta_{set}=0.01{}^{\circ}$ (Case 1) to 4.14 N at $\theta_{set}=0.02{}^{\circ}$ (Case 2) and further to 6.22 N at $\theta_{set}=0.03{}^{\circ}$ (Case 3), demonstrating the expected twofold and threefold scaling. The corresponding measured web-edge displacement $y_{L}$ also followed this proportionality, increasing from 0.046 mm to 0.102 mm and 0.165 mm across the same cases. Additionally, the equivalent misalignment angle ($\theta_{eq}$) exhibited a near-linear dependence on the side-to-side tension difference (ΔT), validating the analytical model that predicts $\theta_{eq}\propto \Delta T$ within small-angle conditions.

The influence of baseline web tension was also consistent with mechanical expectations. For a given $\theta_{set}$, higher web tensions resulted in larger ∆T, as even small misalignments generated stronger imbalance in tension. Conversely, the associated $y_{L}$ decreased with increasing tension, reflecting the greater stiffness of the web that suppressed lateral displacement. For example, at $\theta_{set}=0.02{}^{\circ}$, ∆T rose from 4.14 N (low tension) to 4.21 N (medium tension) and 4.35 N (high tension) while $y_{L}$ reduced from 0.102 mm to 0.109 mm and 0.118 mm, respectively.

Regarding prediction accuracy, the reconstructed displacements closely matched measurements across all test cases, with $RE_{y}$ consistently within 2–3%. The reconstructed equivalent angles $\theta_{eq}$ also exhibited stable accuracy, maintaining $RE_{\theta }$ values within approximately 4–6% regardless of operating tension level. These results demonstrate that the proposed diagnostic framework maintains robustness across a range of operating conditions, validating its capability to generalize beyond a single test configuration. The reference case in Section 4.1 demonstrated the feasibility of using side-to-side tension imbalance to predict lateral displacement and equivalent misalignment angle. To confirm that this diagnostic framework generalizes beyond a single operating point, validation was performed across a matrix of nine test cases, combining three tension levels (low, medium, and high) with three imposed roll misalignment angles (0.01°, 0.02°, 0.03°).

### 4.3. Parameter-Dependent Behavior of Tension-Based Estimation

While Section 4.2 established the general validity of the proposed diagnostic framework across multiple web tensions and imposed misalignment angles, a closer examination reveals how individual operating parameters influence the diagnostic response. This subsection interprets the experimental findings from a mechanistic perspective, emphasizing the role of baseline tension and misalignment angle on both the differential tension response and the reconstructed displacement.

First, the influence of web tension was found to be twofold. At higher baseline tensions, even small angular offsets induced larger side-to-side tension differences ∆T, reflecting the increased force required to maintain lateral equilibrium. Conversely, the corresponding web-edge displacement decreased with increasing tension, since the higher tensile stiffness of the web suppressed lateral deflection. This trade-off highlights that high-tension operation amplifies the diagnostic signal ∆T, while simultaneously reducing the measurable displacement, providing a favorable condition for robust estimation.

Second, the imposed misalignment angle directly governed the scaling of both ∆T and $y_{L}$. Within the tested range (0.01°, 0.02°, 0.03°), the relationship remained linear, confirming that the equivalent-angle formulation is valid in the small-angle regime. However, the proportional increase of $y_{L}$ with $\theta_{eq}$ indicates that larger offsets yield stronger and more easily detectable diagnostic signatures, albeit at the cost of higher lateral deviation in practice. This suggests that the method is particularly effective during early alignment checks, where moderate offsets can be intentionally introduced to calibrate the diagnostic response.

Finally, the interaction of tension level and misalignment angle defines the effective operating window of the approach. The most pronounced tension imbalance occurred under high-tension, large-angle combinations, whereas low-tension, small-angle conditions produced weaker diagnostic signals that approached the sensitivity limit of the sensors. These observations indicate that practical deployment should carefully consider both baseline tension settings and allowable alignment tolerances to ensure accurate and stable estimation. These results collectively demonstrate that operating parameters not only influence the magnitude of tension imbalance but also define the diagnostic robustness of the proposed method.

### 4.4. Sensitivity to Tension Hunting

In practical R2R processing, tension hunting is an unavoidable phenomenon arising from actuator dynamics, drive oscillations, or feedback loop interactions. These fluctuations, typically manifested as low-frequency oscillations superimposed on the nominal tension, directly perturb the side-to-side tension difference (ΔT) that underpins the proposed diagnostic framework. As ΔT serves as the primary indicator of misalignment, any periodic disturbance can propagate into the estimated lateral displacement and equivalent misalignment angle.

**Figure 7:**
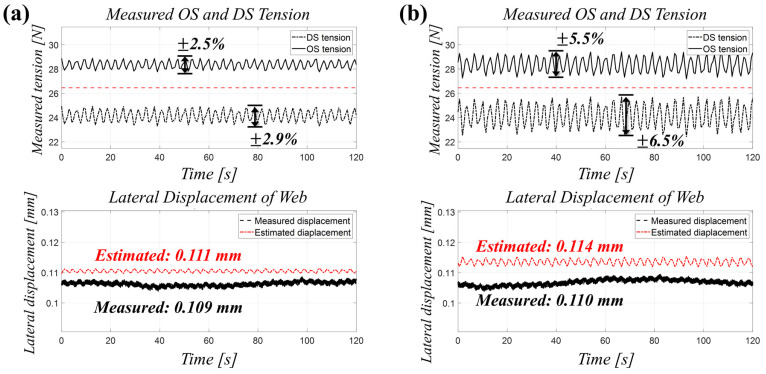
(**a**) Acquired OS and DS tension data and (**b**) estimated misalignment of web under high-level tension hunting.

To evaluate the diagnostic model’s performance under varying levels of tension hunting, we compared the ±2.7% hunting observed in Case 5 with a controlled case featuring a higher hunting level of ±6.0%. As detailed in Figure 7 and Table 5, the mean differential tension ($\Delta T_{mean}$) increased from 4.21 N in the lower hunting case to 4.68 N in the higher hunting case. This rise in tension hunting directly led to increased estimation errors for both lateral displacement and the equivalent misalignment angle, demonstrating the model’s sensitivity to greater tension fluctuations.

Quantitatively, the relative error for displacement ($RE_{y}$) rose from 1.8% to 4.6%, while the relative error for the equivalent misalignment angle (${RE}_{\theta }$) increased from 5.0% to 8.5%. Although a slight increase in error was observed, the diagnostic accuracy was largely maintained, demonstrating the model’s robustness under typical disturbance conditions. The additional error remained within a few percent of the mean displacement and equivalent misalignment angle, indicating the method’s reliability in a production environment.

In conclusion, this analysis confirms that while tension hunting introduces minor perturbations into the estimation process, its overall impact remains limited relative to the magnitude of the misalignment-induced signal. With the implementation of appropriate filtering or system-level tuning, the proposed diagnostic method can be applied reliably in a production environment where disturbance rejection is critical.

## 5. Conclusions

### 5.1. Summary of Key Findings

This study successfully demonstrates a novel, sensor-efficient approach for the real-time diagnosis of roll misalignment in industrial-scale R2R systems. We established a direct, physically grounded correlation between the side-to-side tension difference (ΔT) and web misalignment. Using standard, readily available OS and DS tension sensors, our method accurately predicted lateral web displacement and the equivalent misalignment angle.

The results validated the model’s performance under various process conditions, including different operating tensions, and web guide roller configurations. A key finding is the robust linear relationship between ΔT and the equivalent misalignment angle, which allows for reliable estimations across a broad range of operating parameters. We further demonstrated that the diagnostic framework is resilient to disturbances like tension hunting as discussed in the previous section.

In essence, this work provides a practical and effective solution that complements conventional methods by reducing the reliance on complex calibration and additional sensors. The proposed ΔT-based diagnostic strategy leverages tension signals that are inherently available in standard roll-to-roll tension-control systems, eliminating the need for auxiliary optical sensors or additional hardware. Because the diagnostic function can be implemented through software integration within existing control units, no geometric calibration or physical modification of the web path is required. Consequently, system complexity and setup time are significantly reduced, allowing rapid deployment of the diagnostic framework during production start-up while ensuring full compatibility with existing industrial architectures. By establishing a foundation for leveraging readily available tension data, this approach further enhances quality control and process stability in the continuous manufacturing of polymer films.

### 5.2. Contributions to Web Handling and Polymer Processing Field

This study advances the field of web handling by demonstrating that side-to-side tension difference, a signal inherently available in most industrial R2R systems, can be directly leveraged for misalignment diagnostics. Unlike conventional beam-based or camber-based analyses that require geometric calibration or additional sensors, our approach extracts alignment information solely from tension measurements already integrated into production lines. This sensor-efficient strategy reduces both system complexity and setup time, thereby addressing one of the persistent barriers to implementing real-time diagnostics in high-throughput manufacturing.

Before discussing the broader implications, it is important to clarify the relationship between the theoretical and experimental components of this study. Equations (10)–(13) provide the theoretical formulation linking side-to-side tension imbalance to web misalignment, whereas Section 4.1, Section 4.2, Section 4.3 and Section 4.4 present independent experimental validation and quantitative error analysis under varied process conditions. This separation ensures that the analytical framework and empirical verification were developed and assessed independently, providing a transparent evaluation of model validity.

From a materials-science standpoint, the proposed tension-based diagnostic framework contributes to maintaining uniform stress distribution across the polymer web, thereby minimizing localized deformation and residual strain accumulation. This stability helps preserve the mechanical and optical properties of polymer substrates during coating, lamination, and drying steps. By preventing tension-induced microstructural distortion, the method supports the consistent formation of functional layers in multilayer devices. Consequently, the present study bridges mechanical diagnostics and materials reliability, offering a pathway to enhance both process stability and material integrity in roll-to-roll manufacturing of polymer films.

For polymer processing, the method offers a practical tool to maintain dimensional stability and surface quality during continuous film transport. By linking lateral displacement directly to tension imbalance, we provide a framework that can be applied to multilayer lamination, coating, and printing processes where precise alignment is critical. The contributions of this work are therefore twofold: (1) it establishes a physically grounded, experimentally validated correlation between misalignment and tension imbalance, and (2) it translates this correlation into a real-time diagnostic tool with direct relevance to industrial polymer film manufacturing

### 5.3. Future Directions

While the present work validates the feasibility of tension-based misalignment estimation, several research extensions are warranted. First, adaptive estimation schemes could be developed to account for dynamic changes such as roll replacement, tension transitions, or variable web properties during long production runs. Integration of adaptive filtering or machine learning techniques may further enhance robustness under fluctuating noise and non-stationary operating conditions.

Second, extending the method to multilayer lamination and printed structures represents an important direction. In such applications, even minor misalignments accumulate across layers, and a tension-based diagnostic could serve as an early indicator to prevent registration drift. Third, validation on non-polymeric webs such as metal foils, paper, or composite substrates would enable cross-material generalization, further broadening industrial applicability.

Finally, coupling this diagnostic approach with feedback-based control schemes could lead to hybrid solutions where misalignment is not only detected but also compensated in real time.

## Figures and Tables

**Table 1:** Material properties of polymeric film (at ambient temperature 22 °C).

Polymer Film (CH34P)	
Thickness	75 μm
Width	150 mm
Young’s modulus	3.19 ± 0.08 GPa
Yield stress	109.7 ± 5 MPa
Poisson’s ratio	0.37
**Processing conditions**	
Operating speed	5 m/min
Operating tension	13.2 N , 26.5 N , 36.7 N

**Table 2:** Experimental case configuration: roll misalignment angle and web tension level.

Case	Tension Level [N]	Misalignment Angle [°]	Description
1	Low (13.2)	0.01	Low-tension, small misalignment
2	Low (13.2)	0.02	Low-tension, medium misalignment
3	Low (13.2)	0.03	Low-tension, severe misalignment
4	Medium (26.5)	0.01	Medium-tension, small misalignment
5	Medium (26.5)	0.02	Baseline medium-tension, medium misalignment
6	Medium (26.5)	0.03	Medium-tension, severe misalignment
7	High (36.7)	0.01	High-tension, small misalignment
8	High (36.7)	0.02	High-tension, medium misalignment
9	High (36.7)	0.03	High-tension, severe misalignment

**Table 3:** Summary of tension difference, predicted displacement, measured displacement, and equivalent misalignment angle under medium-tension condition ($T=26.5\;N,\theta_{set}=0.02{}^{\circ}$).

Parameter	Symbol	Estimated	Measured	Reference
		(Proposed Method)	(Displacement Sensor)	(EPC Imposed)
side-to-side tension difference	∆T	Directly obtained	-	-
Web edge displacement	$y_{L}$	From Equations (12) and (13)	Ultrasonic sensor	-
Equivalent misalignment angle	$\theta_{eq}$	Converted via Equation (11)	-	$\theta_{set}=0.02{}^{\circ}$

**Table 4:** Case-wise summary of validation results.

Case	Mean ΔT [N]	$\mathrm{Estimated}\;y_{L}$ [mm]	$\mathrm{Measured}\;y_{L}$ [mm]	$\mathrm{Predicted}\;\theta_{eq}$ [°]	$RE_{y}$ [%]	$RE_{\theta }$ [%]
1	2.06 ± 0.04	0.047 ± 0.002	0.046 ± 0.002	0.0105 ± 0.0005	2.2 ± 0.4	5.0 ± 0.6
2	4.14 ± 0.6	0.105 ± 0.003	0.102 ± 0.003	0.0210 ± 0.001	2.9 ± 0.5	5.0 ± 0.6
3	6.22 ± 0.09	0.154 ± 0.005	0.165 ± 0.006	0.0316 ± 0.001	6.7 ± 1.2	5.3 ± 0.8
4	2.19 ± 0.04	0.055 ± 0.002	0.054 ± 0.002	0.0104 ± 0.0005	1.9 ± 0.4	4.0 ± 0.6
5	4.21 ± 0.06	0.111 ± 0.003	0.109 ± 0.003	0.0210 ± 0.001	1.8 ± 0.4	5.0 ± 0.6
6	6.32 ± 0.10	0.164 ± 0.006	0.172 ± 0.006	0.0317 ± 0.001	4.7 ± 0.9	5.7 ± 0.8
7	2.16 ± 0.04	0.049 ± 0.002	0.050 ± 0.002	0.0104 ± 0.0005	2.0 ± 0.4	4.0 ± 0.6
8	4.35 ± 0.07	0.121 ± 0.004	0.118 ± 0.004	0.0209 ± 0.001	2.5 ± 0.5	4.5 ± 0.7
9	6.52 ± 0.11	0.176 ± 0.006	0.185 ± 0.007	0.0314 ± 0.001	4.9 ± 0.9	4.7 ± 0.7

**Table 5:** Comparison of estimated displacement and equivalent misalignment with varying tension hunting level.

Tension Hunting [%]	Mean ΔT [N]	$\mathrm{Estimated}\;y_{L}$ [mm]	$\mathrm{Measured}\;y_{L}$ [mm]	$\mathrm{Predicted}\;\theta_{eq}$ [°]	$RE_{y}$ [%]	$RE_{\theta }$ [%]
±2.7%	4.21 ± 0.06	0.111 ± 0.003	0.109 ± 0.003	0.0210 ± 0.001	1.8 ± 0.4	5.0 ± 0.6
±6.0%	4.68 ± 0.12	0.114 ± 0.005	0.110 ± 0.004	0.0217 ± 0.001	4.6 ± 0.9	8.5 ± 1.3

## Data Availability

Data sets generated during the current study are available from the corresponding author on reasonable request.

## Abbreviations

The following abbreviations are used in this manuscript:

R2R	Roll-to-Roll
OS/DS	Operator/Driver Side
EPC	Edge Position Control
PET	Polyethylene Terephthalate
NI	National Instruments
DAQ	Data Acquisition
RE	Relative Error
